# Variation in organ‐specific *PIK3CA* and *KRAS* mutant levels in normal human tissues correlates with mutation prevalence in corresponding carcinomas

**DOI:** 10.1002/em.22110

**Published:** 2017-07-29

**Authors:** Barbara L. Parsons, Karen L. McKim, Meagan B. Myers

**Affiliations:** ^1^ Division of Genetic and Molecular Toxicology U.S. Food and Drug Administration, National Center for Toxicological Research Jefferson Arkansas

**Keywords:** biomarker, cancer susceptibility, tumor heterogeneity, personalized medicine, clonal selection

## Abstract

Large‐scale sequencing efforts have described the mutational complexity of individual cancers and identified mutations prevalent in different cancers. As a complementary approach, allele‐specific competitive blocker PCR (ACB‐PCR) is being used to quantify levels of hotspot cancer driver mutations (CDMs) with high sensitivity, to elucidate the tissue‐specific properties of CDMs, their occurrence as tumor cell subpopulations, and their occurrence in normal tissues. Here we report measurements of *PIK3CA* H1047R mutant fraction (MF) in normal colonic mucosa, normal lung, colonic adenomas, colonic adenocarcinomas, and lung adenocarcinomas. We report *PIK3CA* E545K MF measurements in those tissues, as well as in normal breast, normal thyroid, mammary ductal carcinomas, and papillary thyroid carcinomas. We report *KRAS* G12D and G12V MF measurements in normal colon. These MF measurements were integrated with previously published ACB‐PCR data on *KRAS* G12D, *KRAS* G12V, and *PIK3CA* H1047R. Analysis of these data revealed a correlation between the degree of interindividual variability in these mutations (as log_10_ MF standard deviation) in normal tissues and the frequencies with which the mutations are detected in carcinomas of the corresponding organs in the COSMIC database. This novel observation has important implications. It suggests that interindividual variability in mutation levels of normal tissues may be used as a metric to identify mutations with critical early roles in tissue‐specific carcinogenesis. Additionally, it raises the possibility that personalized cancer therapeutics, developed to target specifically activated oncogenic products, might be repurposed as prophylactic therapies to reduce the accumulation of cells carrying CDMs and, thereby, reduce future cancer risk. Environ. Mol. Mutagen. 58:466–476, 2017. © 2017 This article is a U.S. Government work and is in the public domain in the USA. Environmental and Molecular Mutagenesis published by Wiley Periodicals, Inc. on behalf of Environmental Mutagen Society

## INTRODUCTION

Large‐scale cancer genome sequencing projects have been valuable in terms of identifying the prevalent cancer driver mutations (CDMs) associated with particular types of cancers [Ciriello et al., 2013; Kandoth et al., 2013; Watson et al., 2013; Stover and Wagle, 2015]. Cancer “drivers” have been defined as genetic events associated with tumor initiation or progression [Alizadeh et al., 2015] and as somatic mutations that increase the fitness of a cell [Fisher et al., 2013]. More broadly, a cancer driver is “a cell autonomous or non‐cell autonomous alteration that contributes to the tumor evolution at any stage—including initiation, progression, metastasis, and resistance to therapy—by promoting a variety of functions including proliferation, survival, invasion, or immune evasion” [Alizadeh et al., 2015]. Some of the most impactful cancer drivers are hotspot point mutations. Hotspot point mutations merit investigation because of their high prevalence in cancer, their established roles as early events in carcinogenesis, and evidence that the mutant proteins can be therapeutic targets and/or determinants of the efficacy of specific anticancer therapies (i.e., biomarkers to inform personalized approaches to cancer treatment) [Lu et al., 2014; Ryan et al., 2015]. There are known hotspot base substitution mutations in the *PIK3CA* and *KRAS* genes. The prevalence of these mutations in carcinomas of the breast, colon, lung and thyroid are provided in Supporting Information Table S1 (data from Catalogue of Somatic Mutations in Cancer, COSMIC) [COSMIC, 2016].

Individual tumors are heterogeneous in terms of histology, genetics, epigenetics, phenotypic markers, and gene expression [Bonavia et al., 2011; Marusyk et al., 2012]. It has been established that cancers exhibit spatial and temporal genetic diversity, meaning different mutations have been detected in different sectors of a cancer and that abundance of clonal subpopulations can evolve over time [Martelotto et al., 2014; Renovanz and Kim, 2014]. Investigations into the genetic heterogeneity of several cancers has enabled clinicians to identify patients who will benefit from particular therapies (i.e., personalized cancer treatment) and, conversely, demonstrated that mutant cell subpopulations can derail the process and drive the development of acquired resistance to therapy [Fisher et al., 2013; Burrell and Swanton, 2014; Stover and Wagle, 2015]. Given the extent of tumor heterogeneity, the prevalence of mutant subpopulations in advanced cancers and the difficulties associated with treating such cancers, it has been suggested that the development of cancer preventative modalities may be a more effective approach for reducing cancer deaths [Maresso et al., 2015].

Because tumor heterogeneity is a consequence and a reflection of the mechanisms driving tumor initiation and progression, understanding the origins of tumor heterogeneity is critical for designing therapeutic strategies to block the development of resistance to treatment [Hiley et al., 2014]. In addition, information about the earliest stages of carcinogenesis is needed as a foundation for experimental approaches to abrogate or delay the development of cancer [Kensler et al., 2016]. For these reasons, there is growing interest in describing the mutations present in normal and pre‐neoplastic tissues and tracking genetic changes during tumor progression and treatment [Hiley et al., 2014; Jamal‐Hanjani et al., 2014; Nakamura et al., 2014; Roberts and Gordenin, 2014; Francioli et al., 2015; Martincorena and Campbell, 2015].

It has been asserted that a majority of cancers (65%, with 95% CI of 39–81%) arise due to spontaneous mutations, a claim based on the correlation between the incidence of cancer in different tissue types and the number of stem cell divisions in those tissues [Tomasetti and Vogelstein, 2015]. Others have claimed that intrinsic factors account for <10 to 30% of lifetime cancer risk and “rates of endogenous mutation accumulation due to intrinsic processes are not sufficient to account for the observed cancer rates” [Wu et al., 2016]. Clearly, additional information about the frequencies of spontaneous mutations in cancer driver genes and the selective advantages they may confer is necessary to clarify how spontaneous mutations contribute to cancer risk and whether reducing spontaneous CDM frequencies through medical intervention is a viable approach for reducing cancer deaths [Martincorena and Campbell, 2015].

Our laboratory uses a high sensitivity method called Allele‐specific Competitive Blocker PCR (ACB‐PCR), to quantify specific CDMs in normal and cancerous tissue samples derived from particular organs [Parsons et al., 2010; Myers et al., 2016, 2015, 2014a]. By comparing ACB‐PCR amplification of unknown samples to that of a standard curve, ACB‐PCR can determine the ratio of mutant:wild‐type sequence in a DNA sample, measuring one specific base substitution mutation at a time, as long as the mutant to wild‐type ratio is ≥10^−5^. The ACB‐PCR limit of detection is considered to be 1 × 10^−5^ because this is the lowest MF standard that can be discriminated experimentally from a wild‐type only control. An ACB‐PCR MF of 10^−5^ equates to the detection of three mutant molecules in a total of 300,000. ACB‐PCR is robust in the measurement of MFs between 10^−1^ and 10^−5^ (ACB‐PCR may underestimate MFs between 10^−1^ and 1). ACB‐PCR can identify robustly which samples have MFs below 10^−5^, but cannot measure MFs below 10^−5^. This sensitivity is unlikely to be sufficient to detect neutral mutations in normal human tissues. For some CDMs, however, this sensitivity has been shown to be sufficient for the mutant quantification in normal rodent and human tissues, likely due to clonal expansion of cells carrying CDMs that confer a positive selective advantage [Parsons et al., 2005, 2010, 2013; McKinzie et al., 2006; Meng et al., 2010a, 2010b 2011; McKinzie and Parsons, 2011; Banda et al., 2015, 2013]. Information about ultralow frequency mutant cell populations can complement existing characterizations of tumor samples by next‐generation sequencing (NGS, which generally detects variant alleles only at frequencies >1% [Cottrell et al., 2014]), to yield a more complete description of the genetic changes present in cancers. Here we report quantification of levels of the *PIK3CA* E545K and H1047R hotspot point mutations in normal colonic mucosa, colonic adenocarcinomas, normal lung, and lung adenocarcinomas, as well as levels of the *PIK3CA* E545K mutation in normal breast, mammary ductal carcinoma, normal thyroid, and papillary thyroid carcinoma (PTC). These mutations were selected because they have the potential to provide a positive selective advantage. The *PIK3CA* E545K (guanine to adenine mutation at codon 545) results in a glutamine to lysine amino acid change in the p110α kinase binding domain. The *PIK3CA* H1047R (adenine to guanine mutation at codon 1047) results in a histidine to arginine amino acid change in the p110α kinase binding domain. Both *PIK3CA* mutations increase activation of PI3K and its binding to the cell membrane, thereby impacting oncogenic transformation, cell proliferation, cell growth, survival, differentiation and altering invasion potential [Myers et al., 2016].

KRAS is a downstream mediator of growth factor receptor signaling, with critical roles in cell proliferation, survival, and differentiation. The *KRAS* G12D (GGT to GAT at codon 12 resulting in a glycine to aspartic acid amino acid change) and *KRAS* G12V (GGT to GTT at codon 12 resulting in a glycine to valine amino acid change) mutations alter the phenotype of KRAS, providing a context‐dependent selective advantage [Parsons and Myers, 2013]. Here, we also report additional measurements of *KRAS* G12D and G12V in normal colonic mucosa. These new data, integrated with that published previously, creates a dataset of four hotspot point mutations (*KRAS* G12D, *KRAS* G12V, *PIK3CA* E545K, and *PIK3CA* H1047R) in normal and malignant tissues from four different organs. Analysis of these data demonstrates that interindividual variability in cancer driver gene mutant fraction (MF) within normal tissues is correlated with the tissue‐specific prevalence of carcinomas carrying the corresponding mutations.

## MATERIALS AND METHODS

### Sample Collection

Procedures for the acquisition and analysis of anonymous human tissues were reviewed by the FDA's IRB (Research Involving Human Subjects Committee, FWA 00006196). The National Disease Research Interchange (NDRI, Philadelphia, PA) and the National Cancer Institute's Cooperative Human Tissue Network (CHTN) were identified as the primary sources of normal and malignant tissues, respectively. Fresh‐frozen, normal breast, normal colonic mucosa, normal lung, and normal thyroid tissue samples, as well as one fresh‐frozen papillary thyroid carcinoma (PTC), were purchased from the NDRI [Parsons et al., 2010; Myers et al., 2016, 2015, 2014a]. Fresh‐frozen, primary breast ductal carcinomas (DCs), colonic adenomas, colonic adenocarcinomas, lung adenocarcinomas, and PTCs, along with normal colonic mucosa samples, were provided by the CHTN [Parsons et al., 2010; Parsons and Myers 2013; Myers et al., 2016, 2015, 2014a]. These samples were collected at autopsy from tissue donors (one sample per donor) who died from causes unrelated to cancer or diseases affecting the relevant target organ. The numbers of samples analyzed for the different mutations are given in Supporting Information Table S2. All tumor specimens were histologically evaluated and classifications were confirmed by board certified pathologists. The extent of information provided regarding the smoking history of the subjects was inconsistent across subjects (and in some instances absent), only allowing for stratification of subjects as ever‐ and never‐smokers.

### DNA Isolation and First‐Round PCR

DNA isolation was performed as described previously [Parsons et al., 2010; Myers et al., 2016, 2015, 2014a], using sufficient tissue to ensure that the characteristic cellular content of each tissue type would be represented proportionally in the genomic DNA. The average weights of the tissue samples processed for breast, colonic mucosa (separated from submucosa), lung and thyroid were 4.0, 0.4, 2.3, and 1.9 g, respectively. A high fidelity, first‐round PCR amplification of a 306 bp sequence encompassing human *PIK3CA* codon 545 was performed for each DNA sample, using 1 µg of EcoRI‐digested genomic DNA in a 200 μl PCR reaction mix of: 10 mM KCl, 10 mM (NH_4_)_2_SO_4_, 20 mM Tris‐HCl (pH 8.75), 2 mM MgSO_4_, 0.1% Triton X‐100, 0.1 mg/ml bovine serum albumin, 0.2 mM dNTPs, and 10 units of cloned *PfuUltra* Hotstart DNA Polymerase (Agilent Technologies, Santa Clara, CA). The primers were 5′‐GGGAAAATGACAAAGAACAG‐3′ and 5′‐AATGTGCCAACTACCAATGT‐3′. The thermocycler reaction conditions were 2 min at 94°C, followed by 31 cycles of 1 min at 94°C, 2 min at 56°C, and 1 min at 72°C, followed by a final 7 min at 72°C extension and 4°C hold.

A high fidelity, first‐round PCR amplification of a 265 bp sequence encompassing human *PIK3CA* codon 1047 was performed for each DNA sample, using 1 µg of EcoRI‐digested genomic DNA in a 200 μl PCR reaction mix of: 10 mM KCl, 10 mM (NH_4_)_2_SO_4_, 20 mM Tris‐HCl (pH 8.75), 2 mM MgSO_4_, 0.1% Triton X‐100, 0.1 mg/ml bovine serum albumin, 0.2 mM dNTPs, and 10 units of cloned *PfuUltra* Hotstart DNA Polymerase (Agilent Technologies). The primers were 5′‐CCAAACTGTTCTTATTACTTATAG‐3′ and 5′‐GAAGATCCAATCCATTTTTGT‐3′. Thermocycler reaction conditions were 2 min at 94°C, followed by 33 cycles of 1 min at 94°C, 1 min at 52°C, and 1 min at 72°C, followed by a final 6 min at 72°C extension and 4°C hold.

The high fidelity, first‐round PCR of a 384 bp sequence encompassing human *KRAS* codon 12 was described previously [Myers et al., 2014a]. Each first‐round PCR employed one intron‐specific primer to avoid amplification of pseudogenes.

### Synthesis of *PIK3CA* and *KRAS* WT and Mutant Standards

Plasmids carrying the mutant or wild‐type sequences (for the two different amplicons of the *PIK3CA* gene) were used as template to synthesize mutant and wild‐type standards. Plasmids carrying G12D mutant *KRAS*, G12V mutant *KRAS*, and wild‐type *KRAS* sequences were used as template to synthesize standards. Each of the standards was synthesized using conditions identical to those used to amplify the unknown genomic DNAs, except that the amount of input plasmid DNA was calibrated prior to PCR to ensure approximately equivalent amplification in the synthesis of standards and unknowns.

### Purification and Quantification of Standards and Unknowns

Purification of *PIK3CA* E545K WT, mutant, and unknown PCR products was accomplished by ion‐pair reverse phase chromatography using non‐denaturing conditions and a Transgenomic WAVE Nucleic Acid Fragment Analysis and Collection system (Omaha, NE). *PIK3CA* H1047R PCR products and *KRAS* PCR products were gel purified as described previously [Myers et al., 2016]. The DNA concentration of each sample was determined using an Epoch Spectrophotometer Model (Biotek, Winooski, VT), calculated as the average of three measurements that varied by ≤10% from the group mean.

### ACB‐PCR

Mutant (either *PIK3CA* E545K, *PIK3CA* H1047R, *KRAS* G12D, or *KRAS* G12V) and WT reference DNA samples were mixed to generate standards with mutant:WT ratios (i.e., MFs) of 10^−1^, 10^−2^, 10^−3^, 10^−4^, 10^−5^, and 0 (containing only the WT sequence), at a concentration of 5 × 10^7^ copies/μl for the *PIK3CA* E545K mutation or 1 × 10^8^ copies/µl for the *PIK3CA* H1047R mutation. Each ACB‐PCR reaction employed 10 μl of each DNA mixture, for a total of 5 × 10^8^ or 1 × 10^9^ copies per reaction for the E545K and H1047R assays, respectively. Each MF standard was analyzed in duplicate, along with a no‐DNA control. ACB‐PCR reactions were performed in 96‐well PCR plates (Thermo Fisher Scientific, Waltham, MA) using three primers: a fluorescein‐labeled mutant‐specific primer (MSP), a non‐extendable blocker primer (BP, to reduce background amplification of WT *PIK3CA*), and an upstream/downstream primer [Myers et al., 2014b]. The ACB‐PCR measurement of *KRAS* G12D and G12V was performed as described previously [Myers et al., 2015].

For *PIK3CA* E545K mutation, each 50 µl ACB‐PCR reaction contained: 1× Universal Taq Buffer [New England BioLabs, Inc. (NEB), Beverly, MA), 1.5 mM MgCl_2_, 0.1 mg/ml gelatin, 1.0 mg/ml Triton X‐100, 80 µM dNTPs, 400 nM each primer [MSP, 5′‐fluorescein‐TCCTCTCTCTGAAATCACCA‐3′; BP, 5′‐TCCTCTCTCTGAAATCACCdG′3′ (i.e., 3′‐deoxyG modification); and downstream primer, 5′‐CAGAGAATCTCCATTTTAGC‐3′], 3 ng/µl ET SSB (NEB), 2.8 mU/µl Perfect Match PCR Enhancer (Stratagene, La Jolla, CA), and 0.08 µl Hemo KlenTaq DNA polymerase (NEB). Cycling conditions were 2 min at 94°C, followed by 36 cycles of 94°C for 30 sec, 41°C for 90 sec, and 68°C for 1 min. The *PIK3CA* E545K ACB‐PCR product is 77 bp in length.

For *PIK3CA* H1047R mutation, each 50 µl ACB‐PCR reaction contained: 1× Universal Taq Buffer, 0.75 mM MgCl_2_, 0.1 mg/ml gelatin, 1.0 mg/ml Triton X‐100, 40 µM dNTPs, 270 nM MSP (5′‐fluorescein‐TGTTGTCCAGCCACCATGTC‐3′), 500 nM BP (5′‐TGTTGTCCAGCCACCATGTdT‐3′), 1.5 µM downstream primer (5′‐GATGCTTGGCTCTGGAATGC‐3′), 1.2 mU/µl Perfect Match PCR Enhancer, and 0.3 µl Hemo KlenTaq DNA polymerase. Cycling conditions were 2 min at 94°C, followed by 41 cycles of 94°C for 30 sec, 49°C for 45 sec, and 72°C for 1 min. The *PIK3CA* H1047R ACB‐PCR product is 148 bp in length. Blocker primers were purchased from Sigma Genosys (The Woodlands, TX), all others were purchased from Integrated DNA Technologies, Inc. (Coralville, IA).

### Vertical Polyacrylamide Gel Electrophoresis, Image Analysis, and Data Collection

Following ACB‐PCR, 10 µl of 6× ficoll loading buffer/dye were added to each 50 µl reaction, and 10 µl of each ACB‐PCR product were analyzed on 8% nondenaturing, polyacrylamide gels. A PharosFX Molecular Imager with an external blue laser (Bio‐Rad) was used to visualize the fluorescent ACB‐PCR products. Quantity One software (Bio‐Rad), with a locally averaged background correction, was used to quantify the pixel intensities of the correct‐sized bands. For the *PIK3CA* E545K, log‐log plots relating MF to fluorescence were constructed and fit with a power function. For the *PIK3CA* H1047R, *KRAS* G12D, and G12V mutations, log‐linear plots relating MF to fluorescence were constructed and fit with a logarithmic function. Using the function of the standard curve and the pixel intensities of the ACB‐PCR products, the MF (ratio of mutant to wild‐type sequence) of each unknown sample was calculated.

### Statistical Analyses

For each sample, MF was calculated as the arithmetic average of three independent ACB‐PCR measurements. The average MF measurement for each sample was log_10_‐transformed. For each mutational target, the geometric mean MF was calculated as the average log_10_‐transformed MF measured for a particular tissue type. Statistical analyses were performed using log_10_‐transformed data.

Log‐transformed datasets were examined for normality using the D'Agostino and Pearson omnibus normality test. Nonparametric approaches were applied to log‐transformed data that were not normally distributed. The Mann‐Whitney rank sum test was used when a quantitative ranking of MFs was possible. Contingency analyses were used to test for statistical significance when datasets contained multiple samples with MFs below the limit of accurate ACB‐PCR quantification (i.e., <10^−5^). Specifically, the numbers of samples with MFs >10^−5^ and <10^−5^ were examined using *χ* ^2^ or Fisher's exact test. Pearson correlation analyses were performed on log‐transformed, normally‐distributed data (Spearman's rank correlation analyses were performed when data were not normally‐distributed). Two‐tailed *P* values <0.05 were considered significant. All statistical analyses were performed using GraphPad Prism 5 Software (GraphPad Software, Inc., La Jolla, CA).

## RESULTS

The goals of this study were to elucidate the role of spontaneous CDMs in tissue‐specific carcinogenesis and to define the prevalence of low frequency CDMs within cancers. Therefore, unlike many studies that examine tumor tissue and normal tumor adjacent sample, this study examined the levels of hotspot CDM in “normal” autopsy samples from men (except breast) and women without cancer or disease in the relevant organ.

Levels of *PIK3CA* E545K mutation (codon 545 GAG→AAG) were measured in normal breast, colonic mucosa, lung, and thyroid DNA samples, as well as in DNA isolated from ductal carcinomas, colonic adenomas, colonic adenocarcinomas, lung adenocarcinomas, and papillary thyroid carcinomas. Each unknown was quantified in three independent experiments, generating a dataset of 441 *PIK3CA* E545K MF measurements. The numbers of samples analyzed for each tissue type are presented in Supporting Information Table S2. Following ACB‐PCR, products were run on vertical polyacrylamide gels. Replicate examples of *PIK3CA* E545K ACB‐PCR output are provided in Supporting Information Figure S1. MF quantification was achieved by interpolation of the fluorescent intensities of unknown samples with that of a standard curve constructed using samples with defined ratios of mutant:WT alleles (i.e., duplicate 10^−1^, 10^−2^, 10^−3^, 10^−4^, 10^−5^, and 0 standards). A representative example of a *PIK3CA* E545K ACB‐PCR standard curve is shown in Supporting Information Figure S2. The average coefficient of determination (*r* ^2^) for the *PIK3CA* E545K standard curves were as follows: breast, 0.9730 (range 0.9662–0.9828); colon, 0.9628 (range 0.9334–0.9831); lung, 0.9595 (range 0.9316–0.9726); and thyroid, 0.9670 (range 0.9595–0.9724). The measured MFs are given in Supporting Information Table S3. The average coefficient of variation for the triplicate *PIK3CA* E545K MF measurements obtained from individual breast, colon, lung, and thyroid samples were 0.36, 0.55, 0.35, and 0.43, respectively.

*PIK3CA* H1047R (codon 1047 CAT→CGT) MF was measured in normal colonic mucosa and lung DNA samples, as well as in DNA isolated from colonic adenomas, colonic adenocarcinomas, and lung adenocarcinomas (see Supporting Information Table S2). Each unknown was quantified in three independent experiments (276 *PIK3CA* H1047R MF measurements). Replicate examples of *PIK3CA* H1047R ACB‐PCR output are provided in Supporting Information Figure S1. A representative example of a *PIK3CA* H1047R ACB‐PCR standard curve is shown in Supporting Information Figure S2. The average coefficient of determination (*r* ^2^) for the standard curves used to measure *PIK3CA* H1047R MF in colon and lung were 0.9849 (range 0.9732–0.9928) and 0.9802 (range 0.9738–0.9840), respectively. The *PIK3CA* H1047R MFs are given in Supporting Information Table S4. The average coefficient of variation for the triplicate *PIK3CA* H1047R MF measurements obtained from individual colon and lung samples were 0.40 and 0.16, respectively.

Fifteen normal colonic mucosa samples were analyzed for *KRAS* G12D (codon 12 GGT→GAT) and G12V (codon 12 GGT→GTT) MFs as described above (90 ACB‐PCR MF measurements), adding to the previously published ACB‐PCR analysis of six colonic mucosa samples [Parsons et al., 2010]. Replicate examples of the *KRAS* G12D and G12V ACB‐PCR output are provided in Supporting Information Figure S1. Representative examples of *KRAS* G12D and G12V standard curves are shown in Supporting Information Figure S2. The average coefficient of determination (*r* ^2^) for the standard curves used to measure *KRAS* G12D and G12V MF in colon were 0.9895 (range 0.9735–0.9969) and 0.9889 (range 0.9744–0.9973), respectively. The *KRAS* G12D and G12V MFs are given in Supporting Information Table S5. The average coefficient of variation for the triplicate *KRAS* G12D and G12V MF measurements obtained from individual colon samples were 0.52 and 0.17, respectively.

### Comparison of *PIK3CA* E545K, *PIK3CA* H1047R, *KRAS* G12D, and *KRAS* G12V MFs Between Normal and Malignant Tissues

**Figure 1:**
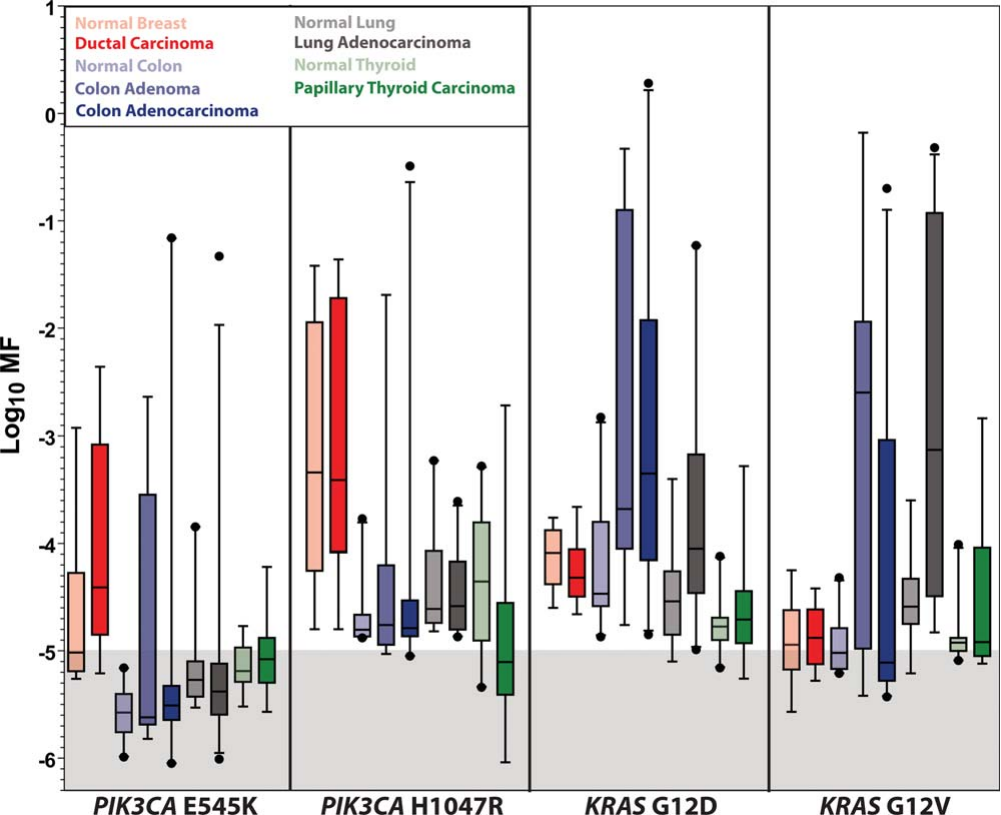
The frequency distributions are shown for *PIK3CA* E545K, *PIK3CA* H1047R, *KRAS* G12D, and *KRAS* G12V in paired datasets of normal and malignant tissues. The data presented include that reported in the current publication, integrated with that reported previously [Parsons et al., 2010; Parsons and Myers, 2013; Myers et al., 2016, 2015, 2014a]. Measurements within the shaded area of the graph are below the lowest MF standard used and are considered below the limit of accurate ACB‐PCR quantification.

A summary of MF measurements for normal tissues and tumors is given in Table 1. No significant differences were observed between the levels of *PIK3CA* E545K mutation in normal tissues and tumor samples, for any of the organs examined (see Fig. 1), although the increased MFs in colonic adenomas compared to normal colonic mucosa were of borderline significance (Fisher's exact test, *P* = 0.0741). No significant differences were observed between the levels of *PIK3CA* H1047R mutation in normal tissue and tumors of the colon or lung (see Fig. 1). For *KRAS* G12D and G12V, the 15 ACB‐PCR measurements from normal colonic mucosa were compared to those previously published for colonic adenomas and adenocarcinomas [Parsons et al., 2010]. This analysis confirmed an earlier report conducted using just six normal samples. Significant differences in MF were observed among sample types (Kruskal‐Wallis test, *P =* 0.0013), with colonic adenomas having significantly greater *KRAS* G12D mutant levels than normal colonic mucosa. Significant differences in *KRAS* G12V MFs were also observed (*χ* ^2^ test, *P =* 0.0220).

**Table 1:** Summary of ACB‐PCR MF Measurements

Sample type	Gene mutation	Tissue	Median MF	Geomean MF	No. samples >10^−5^	No. samples <10^−5^	Mutation positive samples (%)
Normal	*PIK3CA* H1047R	Colon	1.57 × 10^−5^	1.98 × 10^−5^	20	0	100
	*PIK3CA* H1047R	Lung	2.45 × 10^−5^	3.96 × 10^−5^	19	0	100
	*PIK3CA* E545K	Breast	*6.06 × 10^*−6*^*	1.78 × 10^−5^	4	6	40
	*PIK3CA* E545K	Colon	*2.67 × 10^*−6*^*	*2.69 × 10^*−6*^*	0	20	0
	*PIK3CA* E545K	Lung	*5.35 × 10^*−6*^*	*6.75 × 10^*−6*^*	3	16	16
	*PIK3CA* E545K	Thyroid	*6.52 × 10^*−6*^*	*7.02 × 10^*−6*^*	5	14	26
	*KRAS* G12D	Colon	2.88 × 10^−5^	4.90 × 10^−5^	15	0	100
	*KRAS* G12V	Colon	*7.66 × 10^*−6*^*	1.08 × 10^−5^	6	9	40
Adenoma	*PIK3CA* H1047R	Colon	1.73 × 10^−5^	4.56 × 10^−5^	8	1	89
	*PIK3CA* E545K	Colon	*2.15 × 10^*−6*^*	*2.38 × 10^*−6*^*	2	6	25
Carcinoma	*PIK3CA* H1047R	Colon	1.63 × 10^−5^	3.46 × 10^−5^	19	1	95
	*PIK3CA* H1047R	Lung	2.54 × 10^−5^	3.48 × 10^−5^	24	0	100
	*PIK3CA* E545K	Breast	3.92 × 10^−5^	8.36 × 10^−5^	7	2	78
	*PIK3CA* E545K	Colon	*3.07 × 10^*−6*^*	*7.89 × 10^*−6*^*	3	17	15
	*PIK3CA* E545K	Lung	*4.17 × 10^*−6*^*	*7.13 × 10^*−6*^*	4	20	8
	*PIK3CA* E545K	Thyroid	*8.39 × 10^*−6*^*	*8.78 × 10^*−6*^*	7	11	39

The median and geomean MFs shown in italics are below the limit of accurate ACB‐PCR quantification (10^−5^).

The data developed in the current study, combined with that previously published [Parsons et al., 2010; Myers et al., 2016, 2015, 2014a], constitutes a data set of more than 1,800 ACB‐PCR MF measurements on 606 different DNA samples. The mutant frequency distributions observed in normal/tumor tissue pairs are presented in Figure 1, for each mutation analyzed. Figure 1 illustrates for the first time: (1) the tissue‐specific differences in ultralow frequency *KRAS* and *PIK3CA* mutation levels, (2) the remarkable prevalence of low‐frequency mutant populations, and (3) in some cases, a suprising overlap in the frequency distributions between normal and tumor.

### MF Measurements in Normal Tissues

**Figure 2:**
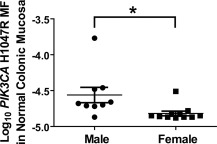
Significantly greater *PIK3CA* H1047R MFs were observed in DNA samples isolated from normal male colonic mucosa as compared to normal female colonic mucosa. The asterisk denotes statistical signficance, Mann Whitney test, *P =* 0.0068.

*PIK3CA* H1047R MF in normal colonic mucosa and lung, along with *PIK3CA* E545K MF in normal colonic mucosa, lung, breast, and thyroid, were examined for correlations with age, smoking status, and gender. No statistically significant associations with age or smoking status were observed (comparing ever smoker vs. never‐smokers). For colonic mucosa, however, the *PIK3CA* H1047R mutation was significantly more abundant in DNA from men than from women (Fig. 2). This novel finding is consistent with the higher age‐adjusted incidence rates for colorectal cancer among men than among women [Abotchie et al., 2012]. In addition, *KRAS* G12D MF in normal colonic mucosa was positively correlated with age, although this correlation was statistically significant at only the 90% confidence level (Spearman *r* = 0.4553, *P =* 0.0576).

**Figure 3:**
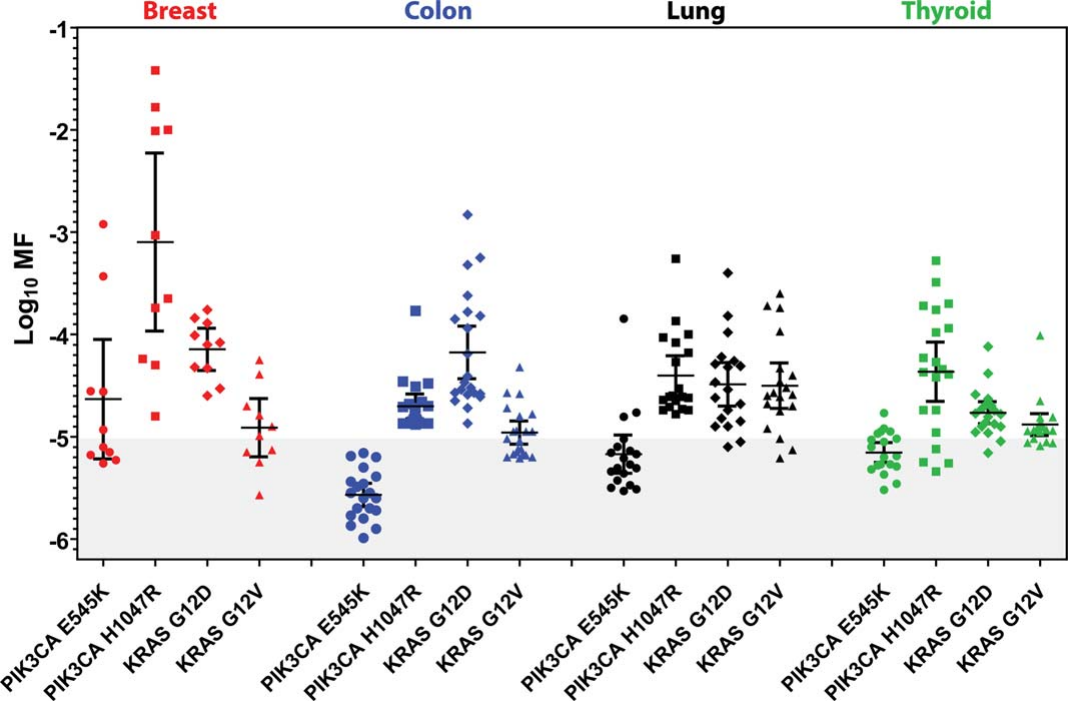
Variability in CDM frequency is tissue‐ and mutant target‐specific. Individual ACB‐PCR measurements were plotted for four different mutations (*PIK3CA* E545K, *PIK3CA* H1047R, *KRAS* G12D, and *KRAS* G12D) in normal DNA samples isolated from four different tissues (breast, colonic mucosa, lung, and thyroid). Measurements within the shaded area of the graph are below the lowest MF standard used and are considered below the limit of accurate ACB‐PCR quantification.

Table 1 and Figure 3 show there are differences in mutation prevalence and variability across normal tissue types. For example, all colonic mucosa samples had *PIK3CA* H1047R and *KRAS* G12D MFs >10^−5^, whereas none of the normal colonic mucosa DNA samples had *PIK3CA* E545K MFs >10^−5^. MF measurements in normal tissues were analyzed statistically for tissue‐specific differences (see Table 1 and Fig. 3). Because many *PIK3CA* E545K mutations were below the limit of accurate ACB‐PCR quantification (10^−5^), statistical differences between normal tissue types were examined using a *χ* ^2^ test (comparing the numbers of samples with MFs greater than and less than 10^−5^ across tissues). This analysis detected a significant difference in levels of *PIK3CA* E545K mutation across the normal tissue types (*P =* 0.0328). Current *PIK3CA* H1047R MF measurements in normal tissues were combined with those published previously. Because all *PIK3CA* H1047R MFs were >10^−5^, analysis of variance was performed using a Kruskal‐Wallis test, with a Dunn's Multiple Comparison post‐test. This analysis identified significant differences in *PIK3CA* H1047R MF across tissue types (*P =* 0.0001), and established that *PIK3CA* H1047R MFs in breast DNA samples were significantly greater than those of DNA from colonic mucosa.

This dataset was used to investigate relationships between tissue‐specific MF measurements and the importance of particular mutations in tissue‐specific carcinogenesis. Frequencies of the *PIK3CA* E545K, *PIK3CA* H1047R, *KRAS* G12D, and *KRAS* G12V mutations in carcinomas of the breast, large intestine, lung, and thyroid were collected from the expertly curated data in the COSMIC database (v76) [COSMIC, 2016] (see Supporting Information Table S1). We determined that the log_10_ geomean MFs from the 16 sets of ACB‐PCR MF measurements collected from normal tissues (*PIK3CA* E545K, *PIK3CA* H1047R, *KRAS* G12D, and *KRAS* G12V in breast, colonic mucosa, lung, and thyroid) were not correlated with the frequencies with which these mutations are reported in carcinomas from each organ (Spearman *r* = 0.3676, *P =* 0.1612).

**Figure 4:**
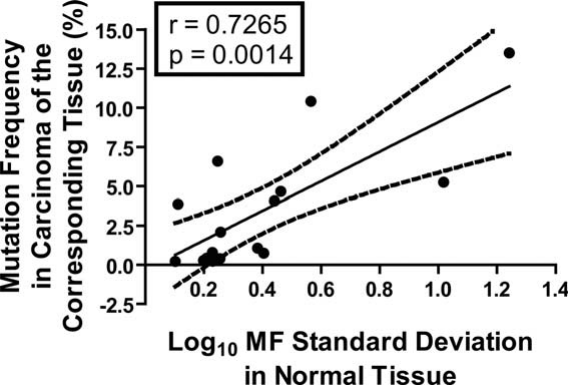
Correlation between the log_10_ MF standard deviation for *PIK3CA* and *KRAS* hotspot point mutations in normal tissues and the frequencies with which the mutations occur in corresponding carcinomas (breast ductal carcinomas, colonic adenocarcinomas, lung adenocarcinomas, and papillary thyroid carcinomas) according to the COSMIC database (v76) [COSMIC, 2016]. Calculated MFs were used, except when calculated MFs were below the theoretical limit of detection 3.33 × 10^−6^ (log_10_ MF = −5.477, or one mutant molecule in a background of 3 × 10^5^ WT molecules).

Visual inspection of the data, however, suggested that mutations reported to occur frequently in particular cancers showed large inter‐individual variability in the ACB‐PCR MFs in normal tissues (e.g., *PIK3CA* H1047R for breast, *KRAS* G12D for colon, see Figs. 1 and 3) [COSMIC, 2016]. Therefore, the relationship between interindividual variability (as log_10_ MF standard deviation) and the frequency of mutation within the various target organs was examined. Calculated MFs were used, except when the calculated MFs were below the theoretical limit of detection 3.33 × 10^−6^ (a log_10_ MF of −5.477), which is equivalent to one mutant molecule in a background of 3 × 10^5^ WT molecules. In order to standardize the analysis of standard deviation across the data set and avoid overestimation of MF standard deviation due to non‐detects, samples with a MF below 3.33 × 10^−6^ were ascribed a MF of 3.00 × 10^−6^ (log_10_ MF −5.481, less than 1 mutant molecule per 300,000). The non‐detects included 2/10 breast *PIK3CA* E545K measurements, 13/20 colon *PIK3CA* E545K measurements, 3/19 lung *PIK3CA* E545K measurements, 1 thyroid *PIK3CA* E454K measurement, and 1 breast *KRAS* G12D measurement. A strong correlation was observed between the log_10_ MF standard deviation and the frequencies of the mutations in carcinomas from the corresponding organs (Spearman *r* = 0.7265, *P =* 0.0014). The correlation was significant with and without the correction for non‐detects, but the correlation was stronger with the correction [Spearman *r* (without correction) = 0.6382, *P =* 0.0078]. Furthermore, the correlation was not driven by a single data point. Even when the breast *PIK3CA* H1047R data point (log_10_ MF standard deviation = 1.2418; COSMIC tumor prevalence = 13.5%) was removed from the analysis, a strong correlation was observed (*r* = 0.6679, *P =* 0.0065). The log_10_ MF variance within normal tissue samples also correlated significantly with the frequency of mutations in tumors of the cognate target organ (Spearman *r* = 0.5088, *P =* 0.0441). In Figure 4, linear regression analysis was used to depict the relationship between log_10_ MF standard deviation in normal tissue and the corresponding prevalence by tumor type for the mutations examined.

## DISCUSSION

The *PIK3CA* and *KRAS* mutations analyzed in this study are recognized hotspot point mutations, meaning they are overrepresented in tumor sequence databases compared to most other mutations. For example, the *PIK3CA* H1047R mutation is the most prevalent somatic mutation detected in DCs. *KRAS* G12D is the most prevalent somatic mutation detected in colonic adenocarcinomas. Beyond that, the signaling dysfunctions caused by the mutant proteins are well‐described. Thus, these mutations are functional drivers of carcinogenesis.

Mutations within these genes are even more prevalent in tumors than previously appreciated, when the mutation detection method employed is capable of detecting mutations within low‐frequency mutant cell subpopulations (see Supporting Information, Table S6). The current study demonstrates *PIK3CA* mutant cells are prevalent as subpopulations within colon adenomas, colon adenocarcinomas, and lung adenocarcinomas. Further, some breast DCs carry subpopulations of *PIK3CA* E545K mutant cells, as well as previously reported subpopulations of *PIK3CA* H1047R [Myers et al., 2016].

We conclude that these particular mutations are able to impact tumor initiation and progression as relatively small subpopulations of cells. An early driver mutation, in a tumor‐initiating clone, is expected to result in a predominant mutation (variant allele frequency of ∼10–50%). Such events would be detected by DNA sequencing and account for the percentages of tumors reported in the COSMIC database (Supporting Information Table S6). The low variant allele frequencies (10^−5^ to 10^−2^), detected in significant fractions of the tumors analyzed, could arise in a number of different ways. First, the mutation could occur late in tumor development. Second, the mutation could occur in an initiating clone, whose growth is outpaced by a cooperating initiating clone or clones. Or, third, the mutation might confer a selective advantage early, but not late in tumor progression. Because these mutations are not sufficient for carcinogenesis, each of these potential paths is expected to be impacted by the accumulation of additional mutations in the tumor mass as a whole.

There is growing evidence that tumors (including breast and colon tumors) can have polyclonal or multiancestral architecture [Parsons, 2008; Zahm et al., 2016]. We propose that some mutations are able to function as “transdriver” mutations, meaning small subpopulations of cells can contribute to (or drive) the initiation and progression of a different clone (or clones) of cells, which carry complementing genetic or epigenetic lesions. Such clonal interactions have been documented [Inda et al., 2011, 2010; Marusyk et al., 2014; Altrock et al., 2015] and are believed to operate through paracrine or non‐cell‐autonomous mechanisms [Hobor et al., 2012]. In a portion of such cases of multiclonal initiation, a *PIK3CA* or *KRAS* mutant cell may acquire an additional genetic hit and progress to become the predominant tumor clone. The alternative pathway is that the co‐initiating clone(s) acquire additional genetic or epigenetic damage and progress to become the predominant tumor clone(s), generating carcinomas with *PIK3CA* or *KRAS* mutant subpopulations.

ACB‐PCR quantification of *PIK3CA* and *KRAS* mutations demonstrated there is a remarkable amount of interindividual variability in the levels of these mutations in some normal tissue types (most notably *PIK3CA* mutations in breast and *KRAS* mutations in colon). Importantly, we report for the first time a significant correlation exists between the degree of interindividual variability in *PIK3CA* and *KRAS* MFs in normal tissues and the tissue‐specific prevalence of the mutations in carcinomas. Conversely, the log_10_ geomean MF did not correlate with the corresponding tumor mutation frequency. Because standard deviation is influenced by the magnitude of the measured variable, the variability among individuals samples combined with the magnitude of the tissue‐specific normal MF measurements may be contributing to the observed correlation (Fig. 4). These observations suggest that some level of mutagenesis (an ACB‐PCR measureable background MF) combined with tissue‐specific clonal expansion (inter‐sample variability in MF measurements) are characteristics of cancer driver mutations within a particular tissue type. These observations have important implications in terms of establishing the relative importance of different mutations as biomarkers of susceptibility for particular human tissues. Further, this suggests that interindividual variation in MF in normal tissues can be used as a metric to assess the context‐dependent, selective advantage of cells carrying oncogenic point mutations.

According to Rozhok and DiGrigori, “oncogenic mutations should have vastly different fitness effects on somatic cells dependent on the tissue microenvironment in an age‐dependent manner” [Rozhok and DeGregori, 2015]. ACB‐PCR MF measurements provide evidence that *PIK3CA* and *KRAS* mutations impact mutant cell fitness in a manner that is context dependent. In the current study, we observed significant differences in *PIK3CA* E545K and H1047R mutation abundance across the different normal tissue types. We also observed a gender difference in *PIK3CA* H1047R MF in colonic mucosa. The significantly greater levels of *PIK3CA* H1047R mutation in male versus female colonic mucosa are consistent with men having a greater risk of developing colon cancer than women. *PIK3CA* H1047R mutation increases in abundance with age in normal breast, suggesting it confers a positive selective advantage in breast [Myers et al., 2016]. *KRAS* G12D and G12V mutations are more abundant in colonic adenomas, as compared to normal colonic mucosa, suggesting these mutations confer a selective advantage early in carcinogenesis. However, there is a significant decrease in *KRAS* G12V during adenoma to adenocarcinoma progression (see Fig. 1) [Parsons et al., 2010], and there is a significant inverse correlation between *KRAS* G12V MF and maximum tumor dimension for colon tumors and papillary thyroid carcinomas [Parsons and Myers, 2013], but not for lung adenocarcinomas [Myers et al., 2015]. These data suggest that *KRAS* G12V is neutral or selected against in some large/advanced cancers. This negative selection in large/advanced cancers could explain the prevalence of pre‐existing *KRAS* mutant tumor cell subpopulations in the colorectal cancers that develop resistance to anti‐EGFR monoclonal antibody therapy [Diaz et al., 2012; Misale et al., 2012].

There are a few reports of *KRAS* mutations being detected in normal colon and lung tissues [Ronai et al., 1994; Yakubovskaya et al., 1995; Sudo et al., 2006; Parsons et al., 2010]. Recently, Gao et al. [2017] reported detecting *KRAS* mutations in 52/156 histologically normal bronchial biopsies from former lung cancer patients. To our knowledge, there is only one report of *PIK3CA* mutation in normal tissue (breast) [Myers et al., 2016], although *PIK3CA* mutations have been detected in breast hyperplasia [COSMIC, 2016] and ductal hyperplasia with columnar cell change [Ang et al., 2012]. Data from the current and previous ACB‐PCR analyses [Parsons et al., 2010; Myers et al., 2016, 2015, 2014a], allow us to conclude that cells carrying *PIK3CA* and *KRAS* hotspot point mutations are prevalent in normal tissues. The prevalence of *PIK3CA* and *KRAS* mutations in normal tissues is consistent with the mathematically‐derived conclusion that “half or more of the somatic mutations in cancers of self‐renewing tissues originate prior to tumor initiation” [Tomasetti et al., 2017, 2013].

There is an ongoing debate about the degree to which spontaneous mutation determines human cancer risk. Recent publications by Tomasetti and Vogelstein highlight the importance of spontaneous mutation in tissue‐specific carcinogenesis [Tomasetti et al., 2013]; they suggest that two‐thirds of cancers are attributable to random mutations in normal stem cells due to replication errors [Tomasetti and Vogelstein, 2015; Tomasetti et al., 2017]. Others contend that “cancer risk is heavily influenced by extrinsic factors,” with only 10 to 30% of cancers due to intrinsic factors [Wu et al., 2016]. Our results refute arguments that spontaneous mutation is insufficiently frequent to have an impact on carcinogenesis, as the combination of spontaneous mutation plus subsequent cell selection must give rise to the relatively high levels of mutations that we observed in normal tissues.

The concept of hotspot transdriver mutations has the potential to unify the opposing schools of thought regarding the importance of intrinsic versus extrinsic factors in carcinogenesis. Spontaneous transdriver mutations can be viewed as substrates that contribute to carcinogenesis, potentially though interaction with exogenously‐induced genetic or epigenetic changes. Our analysis does not discriminate spontaneous from exogenously‐induced mutation. Given the deficits in the smoking histories for the normal tissue samples, the lack of significant associations between MF and smoking history should be interpreted cautiously and revisited in the future. Nevertheless, given the relative insensitivity of epidemiological studies and the relatively small sample sizes analyzed by ACB‐PCR, it seems unlikely that the significant correlation we observed between inter‐individual variability in cancer driver MF and tissue‐specific prevalence of the corresponding mutations in carcinomas is due solely to exogenous exposures. That said, the correlation depicted in Figure 4 may provide some insights into which cancers are impacted by exogenous exposures. The three data points where the cancer incidence is greater than the linear regression expectation correspond to *PIK3CA* E545K, *KRAS* G12D, and *KRAS* G12V in colorectal cancer, a cancer whose incidence is considered to have an extrinsic exposure component.

The remarkable prevalence of *PIK3CA* and *KRAS* mutations in DNA from normal tissues suggests that there may be significant opportunities for cancer prevention. Therapies that target pre‐existing oncomutations in normal tissues have the potential to decrease future cancer risk. Progress is being made in developing therapeutics that target mutant KRAS [Patricelli et al., 2016]. Therapies that target mutant KRAS or PIK3CA proteins could also be investigated as prophylactic therapies in individuals with Noonan and Cowden syndromes, who have high cancer risk because they carry germline mutations in these genes.

## AUTHOR CONTRIBUTIONS

B.L.P. and M.B.M. designed the study. K.L.M., M.B.M., and B.L.P. performed the experiments. B.L.P. prepared figures, tables and manuscript. All authors approved the final manuscript.

## Abbreviations

ACB‐PCR: allele‐specific competitive blocker‐polymerase chain reaction
CDMs: cancer driver mutations
DC: ductal carcinoma
MF: mutant fraction
NGS: next‐generation sequencing
PTC: papillary thyroid carcinoma
WT: wild‐type.

## Supporting information

Supporting Information

## References

[em22110-bib-0001] Abotchie PN , Vernon SW , Du XL. 2012 Gender differences in colorectal cancer incidence in the United States, 1975–2006. J Womens Health 21:393–400. 10.1089/jwh.2011.2992 PMC3321677 22149014

[em22110-bib-0002] Alizadeh AA , Aranda V , Bardelli A , Blanpain C , Bock C , Borowski C , Caldas C , Califano A , Doherty M , Elsner M , et al. 2015 Toward understanding and exploiting tumor heterogeneity. Nat Med 21:846–853. 26248267 10.1038/nm.3915 PMC4785013

[em22110-bib-0003] Altrock PM , Liu LL , Michor F. 2015 The mathematics of cancer: Integrating quantitative models. Nat Rev Cancer 15:730–745. 26597528 10.1038/nrc4029

[em22110-bib-0004] Ang DC , Corless C , Troxell M. 2012 PIK3CA mutation analysis in breast lesions using LNA‐PCR sequencing. J Mol Diagn 14:729 10.1016/j.jmoldx.2012.12.005 23541593

[em22110-bib-0005] Banda M , McKim KL , Haber LT , MacGregor JA , Gollapudi BB , Parsons BL. 2015 Quantification of Kras mutant fraction in the lung DNA of mice exposed to aerosolized particulate vanadium pentoxide by inhalation. Mutat Res 789:53–60. 10.1016/j.mrgentox.2015.07.003 26232258

[em22110-bib-0006] Banda M , Recio L , Parsons BL. 2013 ACB‐PCR measurement of spontaneous and furan‐induced H‐ras Codon 61 CAA to CTA and CAA to AAA mutation in B6C3F1 mouse liver. Environ Mol Mutagen 54:659–667. 24038307 10.1002/em.21808

[em22110-bib-0007] Bonavia R , Inda MDM , Cavenee WK , Furnari FB. 2011 Heterogeneity maintenance in glioblastoma: A social network. Cancer Res 71:4055–4060. 21628493 10.1158/0008-5472.CAN-11-0153 PMC3117065

[em22110-bib-0008] Burrell RA , Swanton C. 2014 Tumour heterogeneity and the evolution of polyclonal drug resistance. Mol Oncol 8:1095–1111. 25087573 10.1016/j.molonc.2014.06.005 PMC5528620

[em22110-bib-0009] Ciriello G , Miller ML , Aksoy BA , Senbabaoglu Y , Schultz N , Sander C. 2013 Emerging landscape of oncogenic signatures across human cancers. Nat Genet 45:1127–1133. 24071851 10.1038/ng.2762 PMC4320046

[em22110-bib-0010] COSMIC . 2016 Catalogue of Somatic Mutations in Cancer. Available at: http://cancer.sanger.ac.uk/cancergenome/projects/cosmic/. Accessed March 2016.

[em22110-bib-0011] Cottrell CE , Al‐Kateb H , Bredemeyer AJ , Duncavage EJ , Spencer DH , Abel HJ , Lockwood CM , Hagemann IS , O'Guin SM , Burcea LC , et al. 2014 Validation of a next‐generation sequencing assay for clinical molecular oncology. J Mol Diagn 16:89–105. 24211365 10.1016/j.jmoldx.2013.10.002 PMC5762937

[em22110-bib-0012] Diaz LA Jr , Williams RT , Wu J , Kinde I , Hecht JR , Berlin J , Allen B , Bozic I , Reiter JG , Nowak MA , et al. 2012 The molecular evolution of acquired resistance to targeted EGFR blockade in colorectal cancers. Nature 486:537–540. 22722843 10.1038/nature11219 PMC3436069

[em22110-bib-0013] Fisher R , Pusztai L , Swanton C. 2013 Cancer heterogeneity: Implications for targeted therapeutics. Br J Cancer 108:479–485. 23299535 10.1038/bjc.2012.581 PMC3593543

[em22110-bib-0014] Francioli LC , Polak PP , Koren A , Menelaou A , Chun S , Renkens I , Van Duijn CM , Swertz M , Wijmenga C , Van Ommen G , et al. 2015 Genome‐wide patterns and properties of de novo mutations in humans. Nat Genet 47:822–826. 25985141 10.1038/ng.3292 PMC4485564

[em22110-bib-0015] Gao W , Jin J , Yin J , Land S , Gaither‐Davis A , Christie N , Luketich JD , Siegfried JM , Keohavong P. 2017 KRAS and TP53 mutations in bronchoscopy samples from former lung cancer patients. Mol Carcinogen 56:381–388. 10.1002/mc.22501 27182622

[em22110-bib-0016] Hiley C , de Bruin EC , McGranahan N , Swanton C. 2014 Deciphering intratumor heterogeneity and temporal acquisition of driver events to refine precision medicine. Genome Biol 15:453–462. 25222836 10.1186/s13059-014-0453-8 PMC4281956

[em22110-bib-0017] Hobor S , Misale S , Crowley E , Scala E , Zanon C , Di Nicolantonio F , Bardelli A. 2012 166 acquired resistance to anti EGFR therapy in colorectal cancer and paracrine protection by KRAS mutated cells. Eur J Cancer 48:51.

[em22110-bib-0018] Inda MDM , Bonavia R , Narita A MY , Sah DWY , Vandenberg S , Brennan C , Johns TG , Bachoo R , Hadwiger P , Tan P , et al. 2011 Tumor heterogeneity is an active process maintained by a mutant EGFR‐induced cytokine circuit in glioblastoma. Genes Dev 24:1731–1745. 10.1101/gad.1890510 PMC2922502 20713517

[em22110-bib-0019] Inda MDM , Bonavia R , Sah DWY , Vandenberg S , Brennan C , Johns TG , Hadwiger P , Tan P , Cavenee WK , Furnari FB. 2010 Tumor heterogeneity in glioblastoma is an active process driven by a mutant EGFR‐induced paracrine circuit. Cancer Res 70:Abstract 3126.

[em22110-bib-0020] Jamal‐Hanjani M , Hackshaw A , Ngai Y , Shaw J , Dive C , Quezada S , Middleton G , de Bruin E , Le Quesne J , Shafi S , et al. 2014 Tracking genomic cancer evolution for precision medicine: The Lung TRACERx Study. PLoS Biol 12:1–7. 10.1371/journal.pbio.1001906 PMC4086714 25003521

[em22110-bib-0021] Kandoth C , McLellan MD , Vandin F , Ye K , Niu B , Lu C , Xie M , Zhang Q , McMichael JF , Wyczalkowski MA , et al. 2013 Mutational landscape and significance across 12 major cancer types. Nature 502:333–339. 24132290 10.1038/nature12634 PMC3927368

[em22110-bib-0022] Kensler TW , Spira A , Garber JE , Szabo E , Lee JJ , Dong Z , Dannenberg AJ , Hait WN , Blackburn E , Davidson NE , et al. 2016 Transforming cancer prevention through precision medicine and immune‐oncology. Cancer Prev Res 9:2–10. 10.1158/1940-6207.CAPR-15-0406 PMC4955753 26744449

[em22110-bib-0023] Lu YC , Yao X , Crystal JS , Li YF , El‐Gamil M , Gross C , Davis L , Dudley ME , Yang JC , Samuels Y , et al. 2014 Efficient identification of mutated cancer antigens recognized by T cells associated with durable tumor regressions. Clin Cancer Res 20:3401–3410. 24987109 10.1158/1078-0432.CCR-14-0433 PMC4083471

[em22110-bib-0024] Maresso KC , Tsai KY , Brown PH , Szabo E , Lippman S , Hawk ET. 2015 Molecular cancer prevention: Current status and future directions. CA Cancer J Clin 65:345–383. 26284997 10.3322/caac.21287 PMC4820069

[em22110-bib-0025] Martelotto LG , Ng CKY , Piscuoglio S , Weigelt B , Reis‐Filho JS. 2014 Breast cancer intra‐tumor heterogeneity. Breast Cancer Res 16:R48. 10.1186/bcr3658 PMC4053234 25928070

[em22110-bib-0026] Martincorena I , Campbell PJ. 2015 Somatic mutation in cancer and normal cells. Science 349:1483–1489. 26404825 10.1126/science.aab4082

[em22110-bib-0027] Marusyk A , Almendro V , Polyak K. 2012 Intra‐tumour heterogeneity: A looking glass for cancer? Nat Rev Cancer 12:323–334. 22513401 10.1038/nrc3261

[em22110-bib-0028] Marusyk A , Tabassum DP , Altrock PM , Almendro V , Michor F , Polyak K. 2014 Non‐cell‐autonomous driving of tumour growth supports sub‐clonal heterogeneity. Nature 514:54–58. 25079331 10.1038/nature13556 PMC4184961

[em22110-bib-0029] McKinzie PB , Delongchamp RR , Chen T , Parsons BL. 2006 ACB‐PCR measurement of K‐ras codon 12 mutant fractions in livers of Big Blue(registered trademark) rats treated with N‐hydroxy‐2‐acetylaminofluorene. Mutagenesis 21:391–397. 17012303 10.1093/mutage/gel041

[em22110-bib-0030] McKinzie PB , Parsons BL. 2011 Accumulation of K‐Ras codon 12 mutations in the F344 rat distal colon following azoxymethane exposure. Environ Mol Mutagen 52:409–418. 21370285 10.1002/em.20644

[em22110-bib-0031] Meng F , Bermudez E , McKinzie PB , Andersen ME , Clewell HJ , Parsons BL. 2010a Measurement of tumor‐associated mutations in the nasal mucosa of rats exposed to varying doses of formaldehyde. Regul Toxicol Pharmacol 57:274–283. 20347909 10.1016/j.yrtph.2010.03.007

[em22110-bib-0032] Meng F , Knapp GW , Green T , Ross JA , Parsons BL. 2010b K‐Ras mutant fraction in A/J mouse lung increases as a function of benzo[a]pyrene dose. Environ Mol Mutagen 51:146–155. 19658153 10.1002/em.20513

[em22110-bib-0033] Meng F , Wang Y , Myers MB , Wong BA , Gross EA , Clewell HJ , Dodd DE , Parsons BL. 2011 P53 codon 271 CGT to CAT mutant fraction does not increase in nasal respiratory and olfactory epithelia of rats exposed to inhaled naphthalene. Mutat Res 721:199–205. 21324376 10.1016/j.mrgentox.2011.02.004

[em22110-bib-0034] Misale S , Yaeger R , Hobor S , Scala E , Janakiraman M , Liska D , Valtorta E , Schiavo R , Buscarino M , Siravegna G , et al. 2012 Emergence of KRAS mutations and acquired resistance to anti‐EGFR therapy in colorectal cancer. Nature 486:532–536. 22722830 10.1038/nature11156 PMC3927413

[em22110-bib-0035] Myers MB , Banda M , McKim KL , Wang Y , Powell MB , Parsons BL. 2016 Breast cancer heterogeneity examined by high‐sensitivity quantification of PIK3CA, KRAS, HRAS, and BRAF mutations in normal breast and ductal carcinomas. Neoplasia 18:253–263. 27108388 10.1016/j.neo.2016.03.002 PMC4840288

[em22110-bib-0036] Myers MB , McKim KL , Meng F , Parsons BL. 2015 Low‐frequency KRAS mutations are prevalent in lung adenocarcinomas. Personalized Med 12:83–98. 10.2217/pme.14.69 PMC5084916 27795727

[em22110-bib-0037] Myers MB , McKim KL , Parsons BL. 2014a A subset of papillary thyroid carcinomas contain KRAS mutant subpopulations at levels above normal thyroid. Mol Carcinogen 53:159–167. 10.1002/mc.21953 22930660

[em22110-bib-0038] Myers MB , McKinzie PB , Wang Y , Meng F , Parsons BL. 2014b ACB‐PCR quantification of somatic oncomutation. Methods Mol Biol 1105:345–363. 24623241 10.1007/978-1-62703-739-6_27

[em22110-bib-0039] Nakamura J , Mutlu E , Sharma V , Collins L , Bodnar W , Yu R , Lai Y , Moeller B , Lu K , Swenberg J. 2014 The endogenous exposome. DNA Repair 19:3–13. 24767943 10.1016/j.dnarep.2014.03.031 PMC4097170

[em22110-bib-0040] Parsons BL. 2008 Many different tumor types have polyclonal tumor origin: Evidence and implications. Mutat Res 659:232–247. 18614394 10.1016/j.mrrev.2008.05.004

[em22110-bib-0041] Parsons BL , Beland FA , Von Tungeln LS , Delongchamp RR , Fu PP , Heflich RH. 2005 Levels of 4‐aminobiphenyl‐induced somatic H‐ras mutation in mouse liver DNA correlate with potential for liver tumor development. Mol Carcinogen 42:193–201. 10.1002/mc.20083 15761837

[em22110-bib-0042] Parsons BL , Manjanatha MG , Myers MB , McKim KL , Shelton SD , Wang Y , Gollapudi BB , Moore NP , Haber LT , Moore MM. 2013 Temporal changes in K‐ras mutant fraction in lung tissue of Big Blue B6C3F1 mice exposed to ethylene oxide. Toxicol Sci 136:26–38. 24029818 10.1093/toxsci/kft190

[em22110-bib-0043] Parsons BL , Marchant‐Miros KE , Delongchamp RR , Verkler TL , Patterson TA , McKinzie PB , Kim LT. 2010 ACB‐PCR quantification of K‐RAS codon 12 GAT and GTT mutant fraction in colon tumor and non‐tumor tissue. Cancer Invest 28:364–375. 20307197 10.3109/07357901003630975

[em22110-bib-0044] Parsons BL , Myers MB. 2013 KRAS mutant tumor subpopulations can subvert durable responses to personalized cancer treatments. Personalized Med 10:191–199. 10.2217/pme.13.1 PMC5115778 27867401

[em22110-bib-0045] Patricelli MP , Janes MR , Li L‐S , Hansen R , Peters U , Kessler LV , Chen Y , Kucharski JM , Feng J , Ely T , et al. 2016 Selective inhibition of oncogenic KRAS output with small molecules targeting the inactive state. Cancer Discov 6:316–329. 26739882 10.1158/2159-8290.CD-15-1105

[em22110-bib-0046] Renovanz M , Kim EL. 2014 Intratumoral heterogeneity, its contribution to therapy resistance and methodological caveats to assessment. Front Oncol 4:142. 24959421 10.3389/fonc.2014.00142 PMC4050363

[em22110-bib-0047] Roberts SA , Gordenin DA. 2014 Hypermutation in human cancer genomes: Footprints and mechanisms. Nat Rev Cancer 14:786–800. 25568919 10.1038/nrc3816 PMC4280484

[em22110-bib-0048] Ronai Z , Luo FC , Gradia S , Hart WJ , Butler R. 1994 Detection of K‐ras mutation in normal and malignant colonic tissues by an enriched PCR method. Int J Oncol 4:391–396. 21566936 10.3892/ijo.4.2.391

[em22110-bib-0049] Rozhok AI , DeGregori J. 2015 Toward an evolutionary model of cancer: Considering the mechanisms that govern the fate of somatic mutations. Proc Natl Acad Sci USA 112:8914–8921. 26195756 10.1073/pnas.1501713112 PMC4517250

[em22110-bib-0050] Ryan MB , Der CJ , Wang‐Gillam A , Cox AD. 2015 Targeting RAS‐mutant Cancers: Is ERK the key? Trends Cancer 1:183–198. 26858988 10.1016/j.trecan.2015.10.001 PMC4743050

[em22110-bib-0051] Stover DG , Wagle N. 2015 Precision medicine in breast cancer: Genes, genomes, and the future of genomically driven treatments. Curr Oncol Rep 17:15. 25708799 10.1007/s11912-015-0438-0 PMC5854397

[em22110-bib-0052] Sudo H , Li‐Sucholeiki XC , Marcelino LA , Gruhl AN , Zarbl H , Willey JC , Thilly WG. 2006 Distributions of five common point mutants in the human tracheal‐bronchial epithelium. Mutat Res 596:113–127. 16458330 10.1016/j.mrfmmm.2005.12.008

[em22110-bib-0053] Tomasetti C , Li L , Vogelstein B. 2017 Stem cell divisions, somatic mutations, cancer etiology, and cancer prevention. Science 355:1330–1334. 28336671 10.1126/science.aaf9011 PMC5852673

[em22110-bib-0054] Tomasetti C , Vogelstein B. 2015 Variation in cancer risk among tissues can be explained by the number of stem cell divisions. Science 347:78–81. 25554788 10.1126/science.1260825 PMC4446723

[em22110-bib-0055] Tomasetti C , Vogelstein B , Parmigiani G. 2013 Half or more of the somatic mutations in cancers of self‐renewing tissues originate prior to tumor initiation. Proc Natl Acad Sci USA 110:1999–2004. 23345422 10.1073/pnas.1221068110 PMC3568331

[em22110-bib-0056] Watson IR , Takahashi K , Futreal PA , Chin L. 2013 Emerging patterns of somatic mutations in cancer. Nat Rev Genet 14:703–718. 24022702 10.1038/nrg3539 PMC4014352

[em22110-bib-0057] Wu S , Powers S , Zhu W , Hannun YA. 2016 Substantial contribution of extrinsic risk factors to cancer development. Nature 529:43–47. 26675728 10.1038/nature16166 PMC4836858

[em22110-bib-0058] Yakubovskaya MS , Spiegelman V , Luo FC , Malaev S , Salnev A , Zborovskaya I , Gasparyan A , Polotsky B , Machaladze Z , Trachtenberg AC. 1995 High frequency of K‐ras mutations in normal appearing lung tissues and sputum of patients with lung cancer. Int J Cancer 63:810–814. 8847139 10.1002/ijc.2910630611

[em22110-bib-0059] Zahm CD , Szulczewski JM , Leystra AA , Olson TJP , Clipson L , Albrecht DM , Middlebrooks M , Thliveris AT , Matkowskyj KA , Washington MK , et al. 2016 Advanced intestinal cancers often maintain a multi‐ancestral architecture. PLoS One 11:e0150170. 26919712 10.1371/journal.pone.0150170 PMC4769224

