# Supplementary material for: Variation in organ‐specific PIK3CA and KRAS mutant levels in normal human tissues correlates with mutation prevalence in corresponding carcinomas
Source: Environ Mol Mutagen. 2017 Jul 29;58(7):466–76. doi: 10.1002/em.22110 (PMC5601221; doi:10.1002/em.22110)
Supplement: Supplementary file 1 — Supporting Information [file EM-58-466-s001.doc]

**Table S1.** Fraction of Tumors with Detectable Mutations in the COSMIC Database*

| Tumor Type | *PIK3CA* Mutation Frequency | *KRAS* Mutation Frequency | Specific Hotspot Mutation (Mutation Frequency) | | | |
| --- | --- | --- | --- | --- | --- | --- |
|  |  |  | *PIK3CA* E545K | *PIK3CA* H1047R | *KRAS*  G12D | *KRAS*  G12V |
| Breast Ductal Carcinoma | 323/1567 (20.61%) | 10/714 (1.4%) | 57/1567 (3.64%) | 185/1567 (11.81%) | 3/714 (0.42%) | 2/714 (0.28%) |
| Breast Carcinoma | 2653/10069  (26.35%) | 30/3589  (0.84%) | 529/10069  (5.25%) | 1360/10069  (13.51%) | 15/3589  (0.42%) | 8/3589  (0.22%) |
| Colon  Adenoma | 0/92 (0.00%) | 216/583 (37.05%) | 0/92 (0.00%) | 0/92 (0.00%) | 77/583 (13.21%) | 68/583 (11.66%) |
| Colon Adenocarcinoma | 146/1010 (14.46%) | 2317/7752 (29.89%) | 44/1010 (4.36%) | 39/1010 (3.86%) | 800/7752 (10.32%) | 501/7752 (6.46%) |
| Colon Carcinoma | 210/1355  (15.50%) | 2462/8107  (30.37%) | 70/1355  (3.86%) | 50/1355  (2.08%) | 844/8107  (10.41%) | 535/8107  (6.60%) |
| Lung Adenocarcinoma | 128/3226 (3.97%) | 2345/14085 (16.65%) | 29/3226 (0.90%) | 38/3226 (1.18%) | 394/14085 (2.80%) | 484/14085 (3.44%) |
| Lung Carcinoma | 296/7965  (3.72%) | 4842/29581  (16.37%) | 85/7965  (1.07%) | 59/7965  (0.74%) | 1203/29581  (4.07%) | 1381/29581  (4.67%) |
| Papillary Thyroid Carcinoma | 21/1121 (1.87%) | 36/2927 (1.23%) | 3/1121 (0.27%) | 3/1121 0.27%) | 12/2927 (0.41%) | 4/2927 (0.14%) |
| Thyroid Carcinoma | 73/1762  (0.41%) | 129/4805  (2.68%) | 5/1762  (0.28%) | 7/1762  (0.40%) | 38/4805  (0.79%) | 11/4805  (0.23%) |

*****Catalogue of Somatic Mutations in Cancer, <http://cancer.sanger.ac.uk/cosmic>, v76.

**Table S2.** Numbers of Samples Analyzed by ACB-PCR

| Sample Type | Number of Samples Characterized* | | | | Reference** |
| --- | --- | --- | --- | --- | --- |
|  | *PIK3CA* E545K | *PIK3CA* H1047R | *KRAS* G12D | *KRAS* G12V |  |
| Breast (normal) | 10 | 10 | 10 | 10 | 28 |
| Breast Ductal Carcinoma | 9 | 9 | 9 | 9 | 28 |
| Colonic Mucosa (normal) | 20 | 20 | 15/6 | 15/6 | 25 |
| Colon Adenoma | 8 | 9 | 15 | 15 | 25 |
| Colon Adenocarcinoma | 20 | 20 | 22 | 22 | 25, 30 |
| Lung (normal) | 19 | 19 | 19 | 19 | 26 |
| Lung Adenocarcinoma | 24 | 24 | 22 | 22 | 26 |
| Thyroid (normal) | 19 | 20 | 20 | 20 | 27, 28 |
| Papillary Thyroid Cancer | 18 | 18 | 17 | 17 | 27 |

*Samples analyzed in the current study are indicated in red. Those reported previously are indicated in black.

** References for previously published MF measurements are provided.

**Figure S1.** Gel Images of Replicate ACB-PCR Analyses of Colon Samples

**
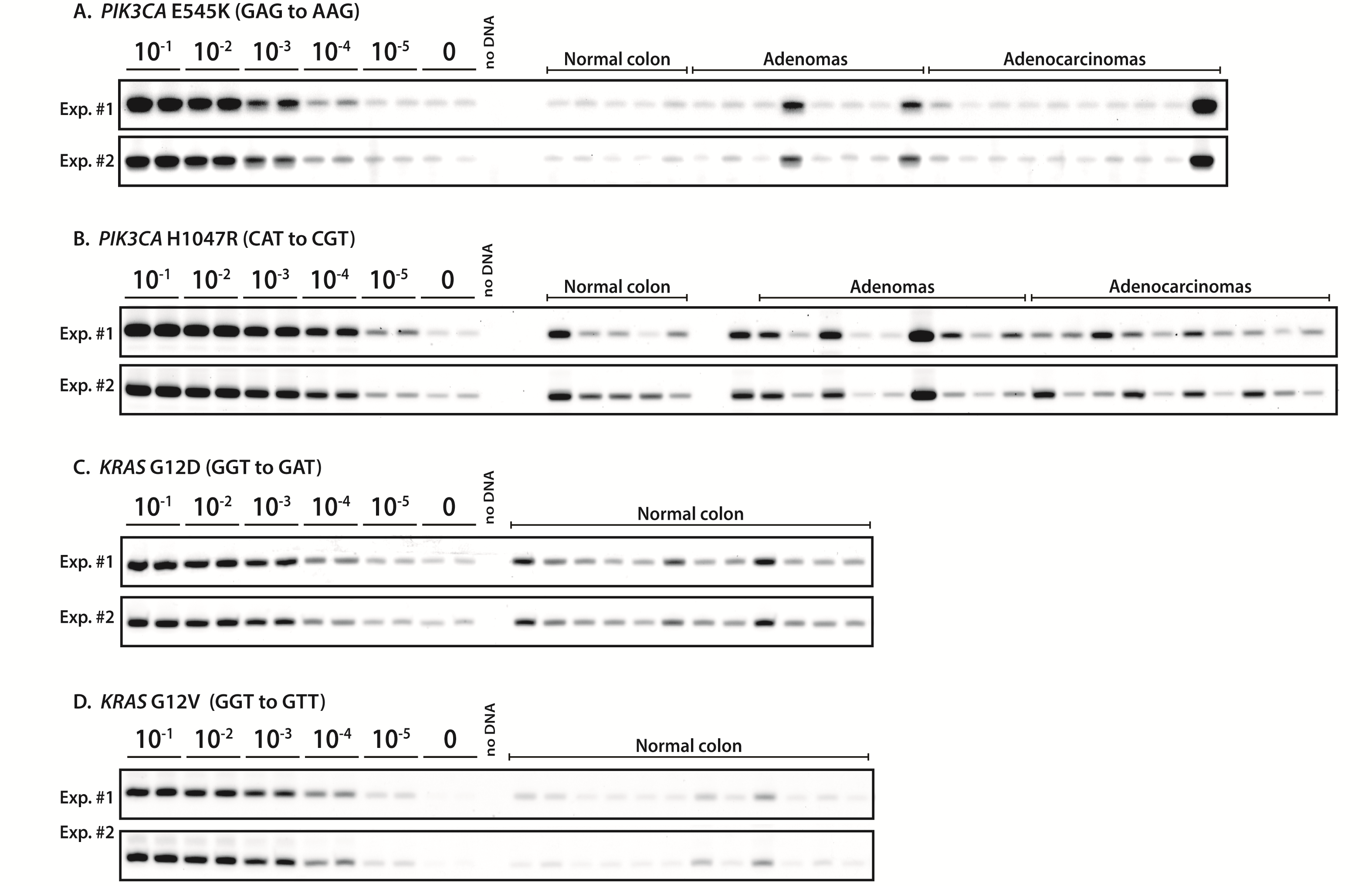
**

**Figure S2.** Standard Curves for ACB-PCR Quantification of MF

**
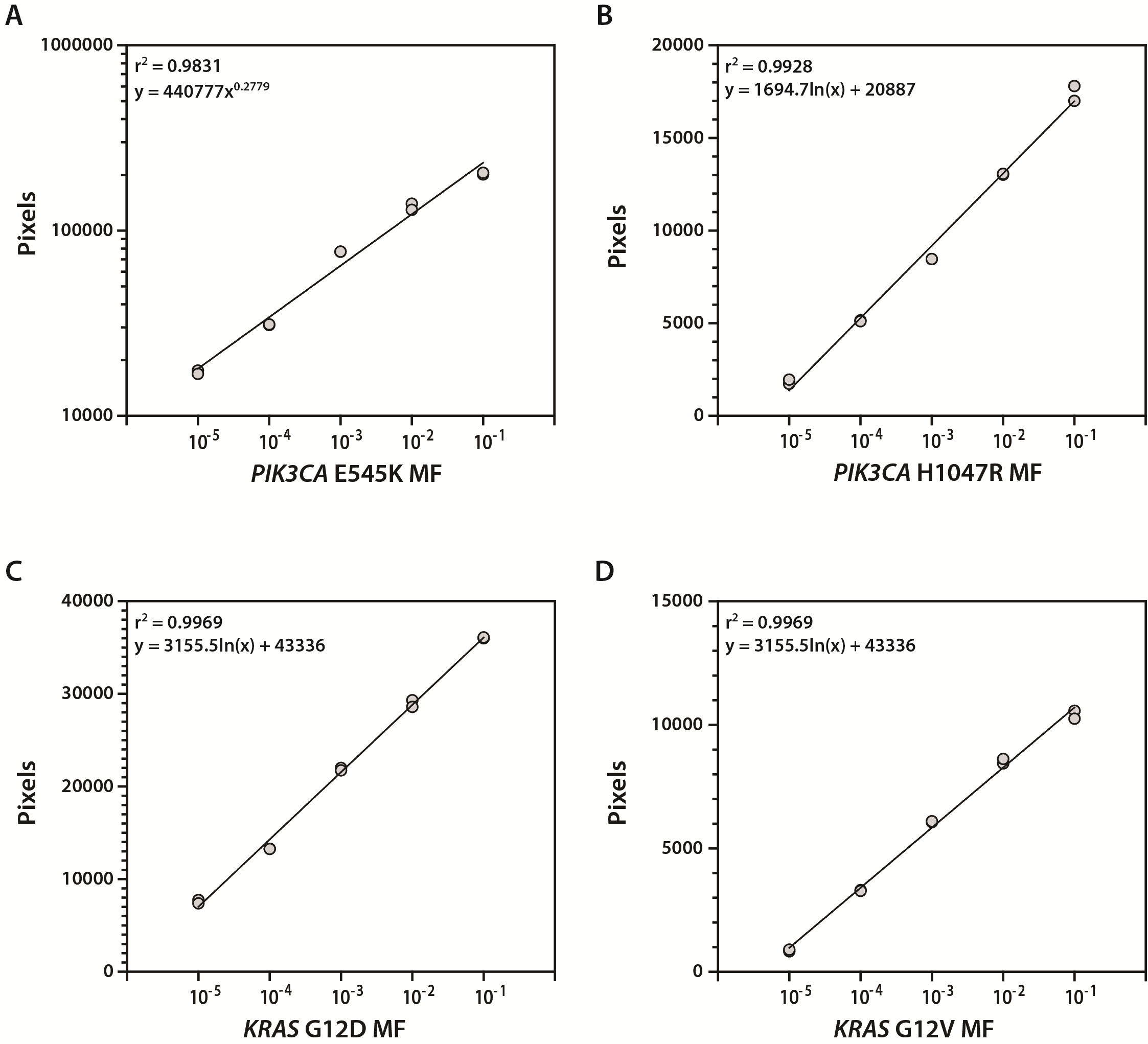
**

**Table S3.** *PIK3CA* E545K MF in Breast, Colon, Lung, Thyroid, and Tumors of those Tissues

| Sample | *PIK3CA* E545K MF in Normal Tissues | | | | *PIK3CA* E545K MF in Tumor Tissues | | | | |
| --- | --- | --- | --- | --- | --- | --- | --- | --- | --- |
|  | Breast* | Colon* | Lung* | Thyroid* | Breast Ductal* CA | Colonic Adenoma* | Colonic AdenoCA* | Lung  AdenoCA* | PTC* |
| 1 | 5.62 x 10^-4^ | *3.43 x 10^-6^* | 1.56 x 10^-5^ | 1.07 x 10^-5^ | 2.01 x 10^-3^ | *2.32 x 10^-6^* | 1.23 x 10^-5^ | *2.65 x 10^-6^* | 1.02 x 10^-5^ |
| 2 | *3.68 x 10^-6^* | *4.05 x 10^-6^* | *3.07 x 10^-6^* | *6.34 x 10^-6^* | 2.73 x 10^-5^ | *7.87 x 10^-6^* | *1.72 x 10^-6^* | *3.03 x 10^-6^* | 1.31 x 10^-5^ |
| 3 | *3.13 x 10^-6^* | *1.35 x 10^-6^* | *7.97 x 10^-6^* | *9.51 x 10^-6^* | 5.16 x 10^-5^ | *1.99 x 10^-6^* | *2.26 x 10^-6^* | *2.50 x 10^-6^* | 1.20 x 10^-5^ |
| 4 | *4.63 x 10^-6^* | *1.92 x 10^-6^* | *4.93 x 10^-6^* | 1.22 x 10^-5^ | *8.25 x 10^-6^* | 2.31 x 10^-3^ | *2.98 x 10^-6^* | *9.83 x 10^-7^* | *9.92 x 10^-6^* |
| 5 | 2.22 x 10^-5^ | *6.43 x 10^-6^* | 1.43 x 10^-4^ | 1.69 x 10^-5^ | 4.34 x 10^-3^ | *1.51 x 10^-6^* | *4.04 x 10^-6^* | *4.60 x 10^-6^* | *2.72 x 10^-6^* |
| 6 | *3.90 x 10^-6^* | *6.28 x 10^-6^* | *6.98 x 10^-6^* | *5.39 x 10^-6^* | 2.37 x 10^-5^ | *2.45 x 10^-6^* | *1.33 x 10^-6^* | *4.12 x 10^-6^* | 1.33 x 10^-5^ |
| 7 | 2.28 x 10^-5^ | *2.83 x 10^-6^* | *3.16 x 10^-6^* | *8.03 x 10^-6^* | *6.13 x 10^-6^* | *2.25 x 10^-6^* | *4.48 x 10^-6^* | 4.65 x 10^-2^ | *2.83 x 10^-6^* |
| 8 | 2.48 x 10^-3^ | *2.49 x 10^-6^* | *4.37 x 10^-6^* | *3.03 x 10^-6^* | 3.45 x 10^-4^ | 9.42 x 10^-4^ | *3.37 x 10^-6^* | *4.21 x 10^-6^* | *4.18 x 10^-6^* |
| 9 | *2.83 x 10^-6^* | *2.81 x 10^-6^* | *2.95 x 10^-6^* | *8.86 x 10^-6^* | 3.92 x 10^-5^ |  | *9.79 x 10^-7^* | *8.79 x 10^-6^* | 6.00 x 10^-5^ |
| 10 | *7.49 x 10^-6^* | *3.66 x 10^-6^* | *9.48 x 10^-6^* | 1.12 x 10^-5^ |  |  | 6.23 x 10^-2^ | 2.84 x 10^-5^ | *8.80 x 10^-6^* |
| 11 |  | *4.98 x 10^-6^* | *6.76 x 10^-6^* | *5.10 x 10^-6^* |  |  | *3.06 x 10^-6^* | *2.32 x 10^-6^* | *7.80 x 10^-6^* |
| 12 |  | *6.91 x 10^-6^* | *7.29 x 10^-6^* | *4.75 x 10^-6^* |  |  | *3.69 x 10^-6^* | *6.38 x 10^-6^* | 1.64 x 10^-5^ |
| 13 |  | *1.68 x 10^-6^* | 1.72 x 10^-5^ | *4.28 x 10^-6^* |  |  | *3.07 x 10^-6^* | *7.36 x 10^-6^* | *7.51 x 10^-6^* |
| 14 |  | *2.01 x 10^-6^* | *6.15 x 10^-6^* | *5.36 x 10^-6^* |  |  | *2.40 x 10^-6^* | *7.58 x 10^-6^* | *7.17 x 10^-6^* |
| 15 |  | *3.22 x 10^-6^* | *3.74 x 10^-6^* | 1.13 x 10^-5^ |  |  | *2.36 x 10^-6^* | *3.13 x 10^-6^* | 2.17 x 10^-5^ |
| 16 |  | *1.03 x 10^-6^* | *4.58 x 10^-6^* | *3.43 x 10^-6^* |  |  | *8.95 x 10^-7^* | *4.44 x 10^-6^* | *4.10 x 10^-6^* |
| 17 |  | *1.60 x 10^-6^* | *5.35 x 10^-6^* | *5.27 x 10^-6^* |  |  | 6.99 x 10^-2^ | *3.08 x 10^-6^* | *5.37 x 10^-6^* |
| 18 |  | *1.25 x 10^-6^* | *3.36 x 10^-6^* | *9.29 x 10^-6^* |  |  | *4.81 x 10^-6^* | *1.66 x 10^-6^* | *7.99 x 10^-6^* |
| 19 |  | *2.52 x 10^-6^* | *4.94 x 10^-6^* | *6.52 x 10^-6^* |  |  | *2.90 x 10^-6^* | 1.29 x 10^-4^ |  |
| 20 |  | *1.98 x 10^-6^* |  |  |  |  | *7.32 x 10^-6^* | *7.60 x 10^-6^* |  |
| 21 |  |  |  |  |  |  |  | 2.46 x 10^-5^ |  |
| 22 |  |  |  |  |  |  |  | *2.05 x 10^-6^* |  |
| 23 |  |  |  |  |  |  |  | *1.82 x 10^-6^* |  |
| 24 |  |  |  |  |  |  |  | *3.07 x 10^-6^* |  |

*The MFs shown in gray italics are below the limit of accurate ACB-PCR quantification (10^-5^).

**Table S4.** *PIK3CA* H1047R MF in Colon, Lung, and Tumors of Colon and Lung

| Sample | *PIK3CA* H1047R MF in Normal Tissue | | *PIK3CA* H1047R MF in Tumor Tissues | | |
| --- | --- | --- | --- | --- | --- |
|  | Colon | Lung | Colonic AdenoCA | Colonic AdenoCA* | Lung AdenoCA |
| 1 | 1.69 x 10^-4^ | 5.46 x 10^-4^ | 4.81 x 10^-5^ | 6.87 x 10^-5^ | 2.03 x 10^-4^ |
| 2 | 2.09 x 10^-5^ | 6.57 x 10^-5^ | 1.30 x 10^-5^ | 1.43 x 10^-5^ | 1.40 x 10^-5^ |
| 3 | 2.17 x 10^-5^ | 2.24 x 10^-5^ | 8.07 x 10^-5^ | 2.91 x 10^-5^ | 1.51 x 10^-5^ |
| 4 | 3.46 x 10^-5^ | 1.34 x 10^-4^ | 1.73 x 10^-5^ | 3.19 x 10^-4^ | 2.08 x 10^-5^ |
| 5 | 1.89 x 10^-5^ | 5.43 x 10^-5^ | *9.23 x 10^-6^* | 1.01 x 10^-5^ | 1.38 x 10^-4^ |
| 6 | 3.28 x 10^-5^ | 2.45 x 10^-5^ | 2.05 x 10^-2^ | 2.80 x 10^-5^ | 1.54 x 10^-4^ |
| 7 | 1.97 x 10^-5^ | 2.39 x 10^-5^ | 3.14 x 10^-5^ | *8.86 x 10^-6^* | 2.35 x 10^-5^ |
| 8 | 1.42 x 10^-5^ | 2.44 x 10^-5^ | 1.01 x 10^-5^ | 2.94 x 10^-5^ | 3.17 x 10^-5^ |
| 9 | 1.36 x 10^-5^ | 1.83 x 10^-5^ | 1.63 x 10^-5^ | 1.13 x 10^-5^ | 1.53 x 10^-5^ |
| 10 | 1.35 x 10^-5^ | 1.01 x 10^-4^ |  | 1.27 x 10^-5^ | 2.74 x 10^-5^ |
| 11 | 1.64 x 10^-5^ | 9.40 x 10^-5^ |  | 1.91 x 10^-5^ | 2.24 x -10^-5^ |
| 12 | 1.32 x 10^-5^ | 1.88 x 10^-5^ |  | 1.34 x 10^-5^ | 2.12 x 10^-5^ |
| 13 | 1.33 x 10^-5^ | 1.95 x 10^-5^ |  | 3.27 x 10^-1^ | 4.45 x 10^-5^ |
| 14 | 3.06 x 10^-5^ | 2.49 x 10^-5^ |  | 4.10 x 10^-5^ | 1.67 x 10^-5^ |
| 15 | 1.49 x 10^-5^ | 1.83 x 10^-5^ |  | 2.14 x 10^-5^ | 1.53 x 10^-5^ |
| 16 | 1.46 x 10^-5^ | 1.66 x 10^-5^ |  | 1.47 x 10^-5^ | 2.25 x 10^-5^ |
| 17 | 1.40 x 10^-5^ | 2.30 x 10^-5^ |  | 1.51 x 10^-5^ | 7.48 x 10^-5^ |
| 18 | 1.36 x 10^-5^ | 8.39 x 10^-5^ |  | 1.66 x 10^-5^ | 5.14 x 10^-5^ |
| 19 | 1.95 x 10^-5^ | 2.96 x 10^-5^ |  | 1.60 x 10^-5^ | 2.96 x 10^-5^ |
| 20 | 1.37 x 10^-5^ |  |  | 1.42 x 10^-5^ | 8.17 x 10^-5^ |
| 21 |  |  |  |  | 5.07 x 10^-5^ |
| 22 |  |  |  |  | 1.73 x 10^-5^ |
| 23 |  |  |  |  | 1.14 x 10^-4^ |
| 24 |  |  |  |  | 1.50 x 10^-5^  * The median and geomean MFs shown in gray italics are below the limit of accurate ACB-PCR quantification (10^-5^). |

*The MFs shown in gray italics are below the limit of accurate ACB-PCR quantification (10^-5^).

**Table S5.** *KRAS G12D and G12V* MF Measurements in Colonic Mucosa

| Sample | *KRAS* MF in Normal Colonic Mucosa | |
| --- | --- | --- |
|  | G12D | G12V* |
| 1 | 5.62 x 10^-4^ | 1.13 x 10^-5^ |
| 2 | 6.44 x 10^-5^ | *9.53 x 10^-6^* |
| 3 | 2.63 x 10^-5^ | *6.35 x 10^-6^* |
| 4 | 1.92 x 10^-5^ | *6.73 x 10^-6^* |
| 5 | 1.34 x 10^-5^ | *6.82 x 10^-6^* |
| 6 | 1.43 x 10^-4^ | *7.03 x 10^-6^* |
| 7 | 3.36 x 10^-5^ | 1.59 x 10^-5^ |
| 8 | 2.55 x 10^-5^ | *7.56 x 10^-6^* |
| 9 | 1.47 x 10^-3^ | 4.74 x 10^-5^ |
| 10 | 2.71 x 10^-5^ | *6.22 x 10^-6^* |
| 11 | 2.88 x 10^-5^ | *7.66 x 10^-6^* |
| 12 | 2.46 x 10^-5^ | *6.37 x 10^-6^* |
| 13 | 3.04 x 10^-5^ | 2.70 x 10^-5^ |
| 14 | 3.93 x 10^-5^ | 1.66 x 10^-5^ |
| 15 | 2.25 x 10^-5^ | 1.93 x 10^-5^ |

*The MFs shown in gray italics is below the limit of accurate ACB-PCR quantification (10^-5^).

**Table S6.** Comparison of Mutation Detection by ACB-PCR and NGS

| Tumor Type | *PIK3CA* Mutation | % Mutant Tumors by ACB-PCR  (mutants/total) | % Mutant Tumors by NGS  (mutants/total) | NGS Variant Frequencies  (lowest ; average)* | Reference for NGS Data |
| --- | --- | --- | --- | --- | --- |
| Colon Adenocarcinoma | E545K | 15  (3/20) | 8.0  (11/138) | 0.06 ; 0.26 | Brannon AR, Vakiani E, Sylvester BE et al. Comparative sequencing analysis reveals high genomic concordance between matched primary and metastatic colorectal cancer lesions. Genome Biology 2014;15:454. |
|  | H1047R | 95  (19/20) | 4.3  (6/138) |  |  |
| Lung Adenocarcinoma | E545K | 16.7  (4/24) | 2.2  (5/230) | 0.06 ; 0.26 | The Cancer Genome Atlas Research Network. Comprehensive molecular profiling of lung adenocarcinoma. Nature 2014;511:543-550. |
|  | H1047R | 100  (24/24) | 0.4  (1/230) |  |  |
| Papillary Thyroid Carcinoma | E545K | 38.9  (7/18) | 0.0  (0/401) | 0.12 ; 0.27 | Agrawal N, Akbani R, Aksoy BA et al. Integrated Genomic Characterization of Papillary Thyroid Carcinoma. Cell;159:676-690. |

*****NGS variant frequencies taken from the TCGA Research Network using cBioPortal [Gao J, Aksoy BA, Dogrusoz U et al. Integrative analysis of complex cancer genomics and clinical profiles using the cBioPortal. Science Signaling 2013;6:pl1; Cerami E, Gao J, Dogrusoz U et al. The cBio Cancer Genomics Portal: An open platform for exploring multidimensional cancer genomics data. Cancer Discovery 2012;2:401-404; <http://www.cbioportal.org/>).
